# Radicalization and Radical Catalysis of Biomass Sugars: Insights from First-principles Studies

**DOI:** 10.1038/srep29711

**Published:** 2016-07-13

**Authors:** Gang Yang, Chang Zhu, Xianli Zou, Lijun Zhou

**Affiliations:** 1 College of Resources and Environments & Chongqing Key Laboratory of Soil Multi-scale Interfacial Process, Southwest University, Chongqing 400715, China

## Abstract

A*b initio* and density functional calculations are conducted to investigate the radicalization processes and radical catalysis of biomass sugars. Structural alterations due to radicalization generally focus on the radicalized sites, and radicalization affects H-bonds in D-fructofuranose more than in D-glucopyranose, potentially with outcome of new H-bonds. Performances of different functionals and basis sets are evaluated for all radicalization processes, and enthalpy changes and Gibbs free energies for these processes are presented with high accuracy, which can be referenced for subsequent experimental and theoretical studies. It shows that radicalization can be utilized for direct transformation of biomass sugars, and for each sugar, C rather than O sites are always preferred for radicalization, thus suggesting the possibility to activate C-H bonds of biomass sugars. Radical catalysis is further combined with Brønsted acids, and it clearly states that functionalization fundamentally regulates the catalytic effects of biomass sugars. In presence of explicit water molecules, functionalization significantly affects the activation barriers and reaction energies of protonation rather than dehydration steps. Tertiary butyl and phenyl groups with large steric hindrances or hydroxyl and amino groups resulting in high stabilities for protonation products drive the protonation steps to occur facilely at ambient conditions.

Petroleum resources are expected to be exhausted in the next few decades, and alternative energy sources are being sought. Owing to the abundance, sustainability and easy availability, cellulosic biomass has been regarded as a promising energy source to replace petroleum^1^,^2^,^3^; nonetheless, the direct transformation of cellulosic biomass to downstream products remains a grand challenge^4^.

Glucose, the monomer of cellulose, has often been used as protype to investigate the transformation of cellulosic biomass^5^,^6^,^7^,^8^,^9^,^10^,^11^,^12^,^13^,^14^,^15^. The Brønsted-acid catalysis of glucose is driven towards the formation of humin precursors and reversion products, while under similar conditions, fructose can be activated readily and with a sequence of facile reaction steps converted to 5-(hydroxymethyl)furfural (HMF) or other platform chemicals^16^,^17^,^18^. Sn-BEA zeolite and HCl solutions were skilfully combined by Nikolla *et al*.^19^, realizing the “one-pot” synthesis of HMF from glucose with relatively high conversion and selectivity. On the other hand, glucose has lower acidity than fructose^20^,^21^,^22^,^23^ and hence is less reactive under basic conditions. Recently, Liu *et al*.^24^ have demonstrated that organic amines are efficient for the isomerization of glucose to fructose. These clearly state that whether in acidic or basic environments, fructose acts as the pivotal intermediate for the transformation of cellulosic sugars.

In the past few decades, radical catalysis has emerged into a flourishing area^25^,^26^, while reports relating to biomass transformations are scarce. Electron magnetic resonances were used to study the irradiation of crystalline D-glucose, D-fructose and sucrose^27^,^28^,^29^,^30^, and several C-centered radicals were assigned with help of density functional calculations^30^,^31^,^32^. The radical stabilities can be expressed using the isodesmic H-transfer reactions^33^,^34^,


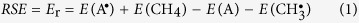


Reaction enthalpy of Eq. 1 is also referred to as radical stabilization energy (*RSE*). The *RSE* calculations with acceptable accuracy allows for quantitative estimation of H-transfer reaction energies and optimization of the properties for new reagents^35^. A comprehensive understanding of C-centered sugar radicals is lacking, which was presently conducted with high-level *ab initio* calculations (MP2/aug-cc-pVTZ). D-glucose (monomer of cellulosic biomass) and D-fructose (pivotal intermediate for cellulose utilization), in both α- and β-anomers, have been considered. To best of our knowledge, all previous sugar conversions focused on O sites, which are preferred over C sites during protonation^16^,^17^,^18^,^36^,^37^,^38^ and deprotonation^20^,^21^,^22^,^23^. Here a systematic study was also conducted for O-centered sugar radicals, and comparisons with the results of C-centered radicals clearly indicated that the formation of C-centered radicals is always preferential, thus suggesting the possibility to activate and convert the C-H bonds of biomass sugars.

Csonka *et al*.^39^ evaluated the performances of different density functionals and basis sets for the various β-D-glucose conformations. However, performances of different density functionals and basis sets for sugar radicals remain elusive, which will be conducted in this work. On such basis, the enthalpy changes and Gibbs free energies for all radicalization processes were presented with high accuracy, which can be referenced for subsequent experimental measurements. Finally, a variety of radical catalytic routes were designed and it clearly demonstrated that radicalization that focuses on activation of C sites can be used for the transformation of biomass sugars; in addition, we found that the presence of explicit water molecules can significantly alter the reaction paths and energies. These are helpful for the transformation of biomass sugars that can solve the global energy crisis.

## Computational Details

**Figure 1:**
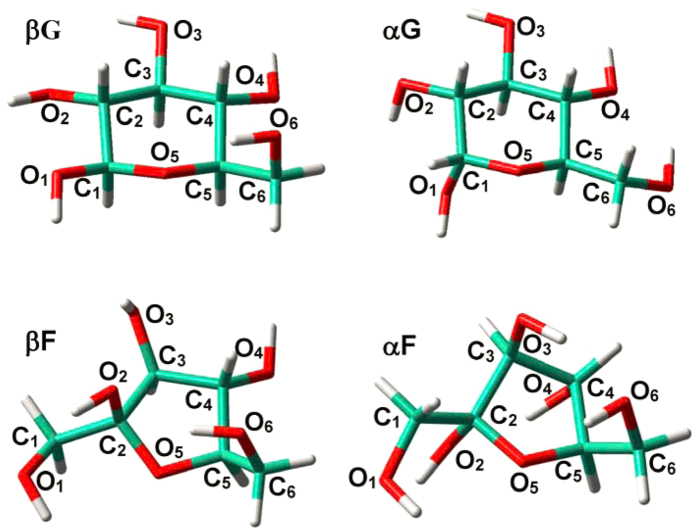
Structures of β-D-glucopyranose (βG), α-D-glucopyranose (αG), β-D-fructofuranose (βF) and α-D-fructofuranose (αF).

In line with previous works^8^,^9^,^10^,^11^,^12^,^13^,^16^,^17^,^18^, the lowest-energy conformers of α,β-D-glucopyranose and α,β-D-fructofuranose were the choice for studies, which were respectively referred to as **αG**, **βG** and **αF**, **βF** (Fig. 1). The various O and C sites potential to be radicalized were identified by atomic numbering; e.g., radicals corresponding to α-D-glucopyranose at O_1_ site and β-D-fructofuranose at C_4_ site were designated to be **αGrO**_**1**_ and **βFrC**_**4**_, respectively. Different electronic states were considered^40^,^41^, and radicals in doublet states that show obviously superior stabilities will be discussed unless otherwise specified.

All calculations were conducted with Gaussian09 suite of programs^42^. Structural optimizations of sugars and their radicals corresponding to the various O/C sites were performed at MP2/aug-cc-pVTZ (denoted as bs4) level^39^. The radicalization process and radical generation energies (Δ*E*_r_) are shown as,


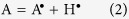



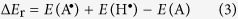


Performances of other functionals and basis sets were then evaluated, using MP2/bs4 to be benchmark as suggested elsewhere^39^: 1) Hartree-Fock (HF)^43^ as well as B3LYP^44^,^45^, BP86^46^, PBE1PBE^47^ and M06L^48^,^49^ density functionals, in combination with the bs4 basis set; 2) Because of relatively fine agreement with MP2 results, B3LYP was further employed with 6-31G(d), 6-31 + G(d, p) and 6-311++G(d, p) (referred to bs1, bs2, and bs3, respectively) to address the effect of basis sets; 3) Single-point energies were run at MP2/bs4 level, on basis of B3LYP optimized structures. The Δ*E*_r_ values of 1)~3) may deviate from those directly from MP2/bs4, and such deviations were defined to be δΔ*E*(*i*) (*i* being the atomic numbering of O/C site). Performances of these functionals and basis sets can be assessed by the average of δΔ*E*(*i*) at the various O/C sites (<δΔ*E*)>) and standard deviations of δΔ*E*(*i*) (*S.D.*).

The enthalpy changes (Δ*H*) and Gibbs free energies (Δ*G*) for the radicalization processes can be given as^20^,


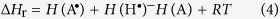



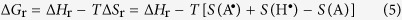


where *S*, *T* and *R* stand for entropy (in terms of tranlational, rotational and vibrational contributions), temperature and gas constant, respectively.

The thermodynamic parameters were calculated at B3LYP/bs2 level and then corrected by δΔ*E*(*i*), which have been testified by a large number of cases to achieve comparable results as those directly from MP2/bs4 method (Tables S1–S4 and Figures S1 and S2),


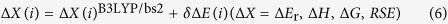


The solvent effects were accounted for by the self-consistent isodensity polarizable continuum model (SCI-PCM) of self-consistent reaction field (SCRF)^50^, in combination with B3LYP/bs2 method. The default dielectric constant (*ε* = 78.4) was used for water solvent.

For catalytic systems, the two-layer ONIOM methodology (MP2/bs4//M06L/bs3)^51^,^52^ that has been validated sufficiently (Tables S1, S5 and Figures S3 and S4) was employed for energy calculations, on basis of B3LYP/bs2 optimized structures. The radicalized C/O sites and neighbouring C/O groups as well as adsorbents (proton and water molecules) were defined as the high-level regions, while the rest were treated as the low-level regions. This methodology can be applied for the accurate energy calculations of other sugar and larger catalytic systems.

## Results and Discussion

### Radicalization of D-glucopyranose

**Figure 2:**
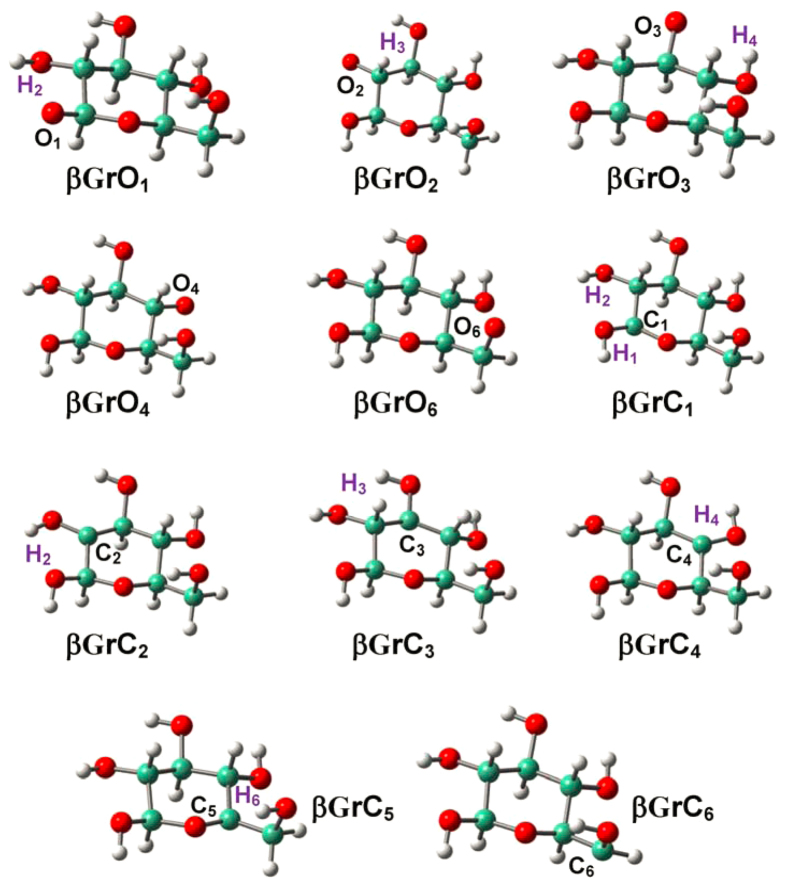
Optimized structures for the radicalization of β-D-glucopyranose (βG) at the various O/C sites.

**Figure 3:**
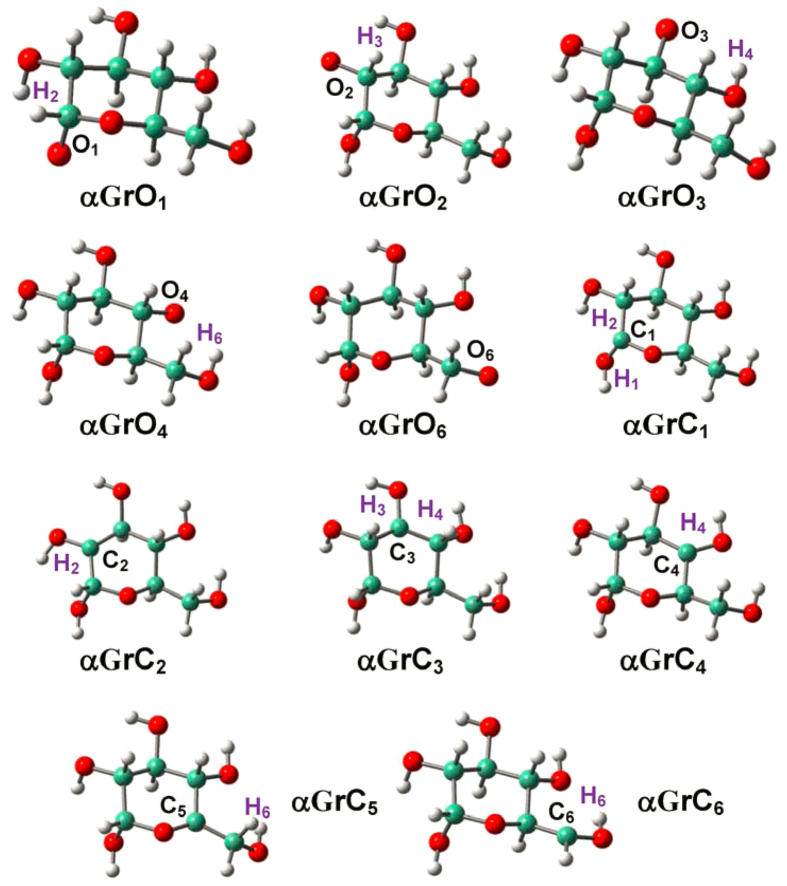
Optimized structures for the radicalization of α-D-glucopyranose (αG) at the various O/C sites.

As indicated in Fig. 1, D-glucopyranose conformers (**βG** and **αG**) are inclined to construct successive H-bonds (O_i+1_H_i+1_…O_i_ type; e.g., O_4_H_4_…O_3_: 2.407 Å, O_3_H_3_…O_2_: 2.436 and O_2_H_2_…O_1_: 2.485 Å in **βG**,)^20^,^53^. Figures 2 and 3 show that no proton migration or ring opening occurs for the radicalization of D-glucopyranose conformers, whether at O or at C sites. Structural alterations are generally small and restricted at the radicalization sites, and two C-centered radicals previously observed during the irradiation of crystalline α-D-glucopyranose show agreement with present results^27^,^28^,^32^.

Radicalization at O sites reinforces associated C-O bonds; e.g., the C_3_-O_3_ bonds are equal to 1.420 and 1.378 Å in **βG** and **βGrO**_**3**_, respectively. The electronic densities on radicalized O atoms decline and thereby proximate H-bonds are impaired; e.g., the Mulliken charges of O_3_ in **βG** and **βGrO**_**3**_ amount to −0.834 and −0.802, causing the elongation of O_4_H_4_…O_3_ H-bond from 2.407 to 2.429 Å. Radicalization at C sites strengthens the associated C-O and C-C bonds, where the pyranyl C-C/C-O bonds are apparently less affected than dangling C-O bonds; e.g., the C_2_-C_3_, C_3_-C_4_ and C_3_-O_3_ bonds are 1.511, 1.513 and 1.420 Å in **βG** and 1.486, 1.486 and 1.363 Å in **βGrC**_**3**_, respectively. The C_3_-O_3_ bond has been corroborated by the delocalized *π*-electron system developed by the lone-pair electrons of O_3_ and half-empty orbital of C_3_, as evidenced by electron transfers from O_3_ to C_3_. Whether in α- or in β-anomer, the hydroxyl (-OH) groups are alternatively up and down with respect to the pyranyl ring that are not beneficial for the formation of O_i+1_H_i+1_…O_i_ type H-bonds, whereas such H-bonds can be substantialized due to radicalization at C sites; e.g., the C_2_C_4_C_3_O_3_ dihedrals are optimized at 122.32° and 143.62° in **βG** and **βGrC**_**3**_, and the larger dihedrals are in favour of O_3_H_3_…O_2_ and O_4_H_4_…O_3_ H-bonds that are 2.436 and 2.407 Å in **βG** while 2.225 and 2.367 Å in **βGrC**_**3**_, respectively. C_2_ site of **αG** is an exception, where the −O_2_H_2_ and −O_1_H_1_ groups fall at the same side of pyranyl ring, and radicalization elongates O_2_H_2_…O_1_ H-bond distance from 2.210 to 2.315 Å.

### Radicalization of D-fructofuranose

**Figure 4:**
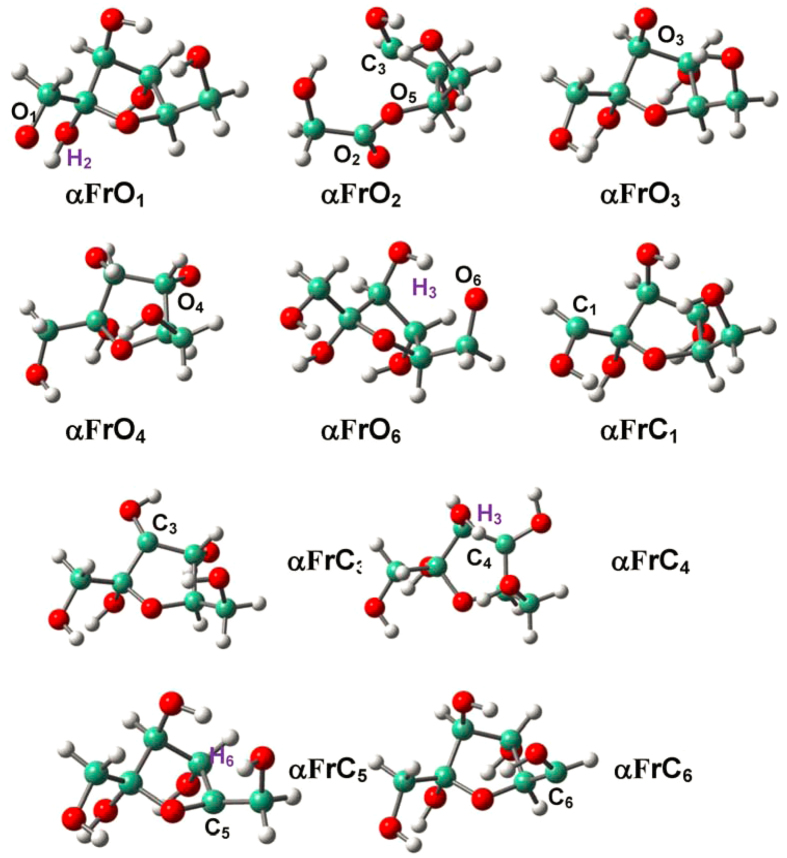
Optimized structures for the radicalization of α-D-fructofuranose (αF) at the various O/C sites.

**Figure 5:**
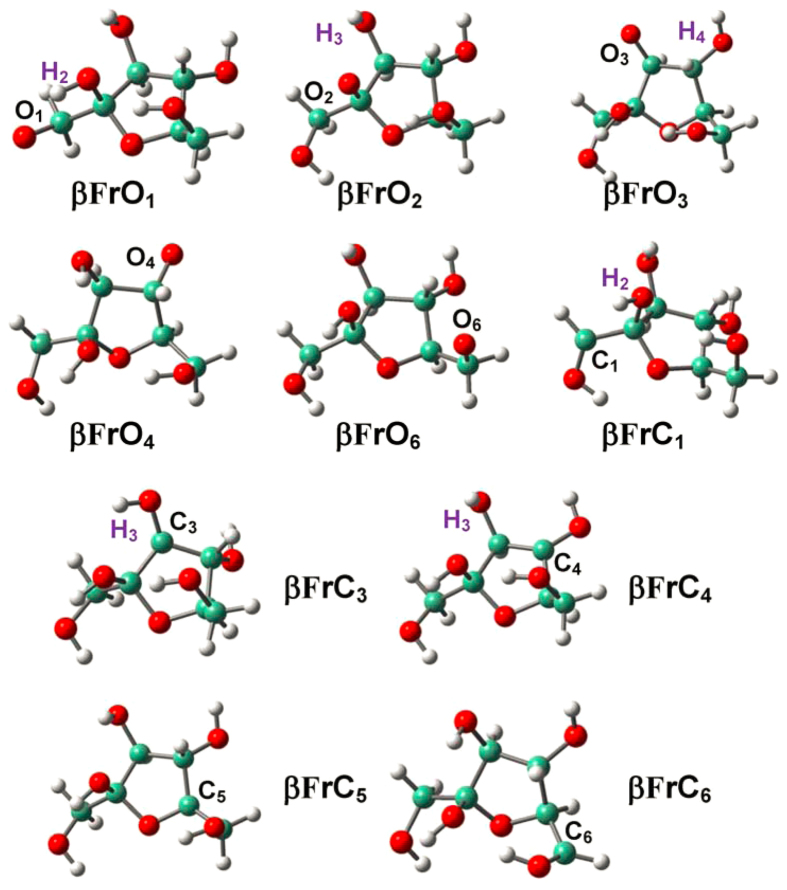
Optimized structures for the radicalization of β-D-fructofuranose (βF) at the various O/C sites.

Radical structures of D-fructofuranose conformers (**αF** and **βF**) are given in Figs 4 and 5. The structural alterations due to radicalization are generally local and focus on radicalization sites, which is in line with the condition of D-glucopyranose conformers^54^. Proton migration/ring opening occurs only at O_2_ site of **αF**, where the furanyl ring opens by breaking C_2_-C_3_ bond with production of the C_2_-O_2_ and C_3_-O_3_ delocalized *π*-electron system.

Radicalization at O sites reinforces dangling C-O bonds and at C sites also strengthens associated furanyl C-C/C-O bonds; meanwhile, proximate H-bonds are elongated, which are consistent with the results of D-glucopyranose conformers; e.g., O_2_H_2_…O_1_ H-bond are 2.211 Å in **αFrO**_**1**_ and 2.191 Å in **αF**. However, the changing trends of H-bonds become elusive for radicalization at C sites as a result of inconsistent roles played by interlaced H-bonds: 1) Shortening as in D-glucopyranose; e.g., O_1_H_1_…O_5_ H-bond: 2.057 Å in **βFrC**_**1**_ vs. 2.602 Å in **βF**; 2) Apparent elongation; e.g., O_2_H_2_…O_1_ H-bond: 2.704 Å in **αFrC**_**1**_ vs. 2.191 Å in **αF**; 3) Construction of new H-bond; e.g., the O_3_H_3_…O_4_ H-bond: 2.449 Å in **αFrC**_**3**_. Radicalization causes associated hydroxyl (-OH) groups to approach the furanyl plane; e.g., the C_2_C_4_C_3_O_3_ dihedrals are 118.98° and −141.97° in **αF** and **αFrC**_**3**_, respectively, and consequently, HO_3_H_3_…O_6_ H-bond is damaged by radicalization of **αF** at C_3_ site, and the −O_3_H_3_ group shifts to the other side of furanyl ring and creates O_3_H_3_…O_4_ H-bond. That is, radicalization at C sites in D-fructofuranose rather than D-glucopyranose conformers result in more pronounced effects on proximate H-bonds.

### Thermodynamic Calculations

The Δ*E*_r_ and *RSE* values of sugars (**βG**, **αG**, **βF** and **αF**) corresponding to the various O/C sites are calculated at MP2/bs4 level (Tables 1 and S4). Eqs 1 and 3 show that they can be correlated with each other,


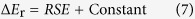


Accordingly, Δ*E*_r_ and *RSE* have exactly the same changing trends. The Δ*E*_r_ data of D-glucopyranose and D-fructofuranose can be comparable, and for each sugar (**βG**, **αG**, **βF** and **αF**), the Δ*E*_r_ values corresponding to the various O/C sites are close to each other, and C rather than O sites are preferred for radicalization, which is consistent with the experimental observations^27^,^28^,^29^,^30^,^31^,^32^. In addition, the Δ*E*_r_ data of two anomers (α/β) can differ substantially, probably as a result of anomeric effects and H-bonding diversities^20^,^53^.

As indicated in Figure S1 and Table S1, density functional methods are apparently superior to HF for treatment of radical sugars and obtain close results as MP2. Then B3LYP, currently one of the most used density functionals, is combined with different basis sets to demonstrate the effect of basis sets. It shows that bs2 and bs3 achieve comparable results with bs4 while bs1 deviates significantly (Figure S2 and Table S1), suggesting that diffuse functions are necessary for treating sugar radicals.

Balancing the computional cost and accuracy, B3LYP/bs2 is selected for further studies, and a number of cases in Tables S2 and S3 demonstrate that the Δ*H* and Δ*G* data calculated at B3LYP/bs2 level deviate remarkably from those of MP2/bs4, while those corrected by energy deviations (δΔ*E*(*i*)) are also reproducible. G4MP2 is probably the most familiar composite method for energy calculations^55^, and other composite methods with zero-point vibrational energies being computed at lower theoretical levels have also been reported^56^,^57^. The Δ*H* and Δ*G* data for radicalization of sugars at the various O/C sites are calculated this way and collected in Table 2. The lowest Δ*H*_r_ and Δ*G*_r_ values for O sites are 466.1 (O_1_) and 381.2 (O_1_) kJ/mol for **βG**, 470.0 (O_1_) and 440.8 (O_1_) kJ/mol for **αG**, 466.4 (O_1_) and 437.2 (O_1_) kJ/mol for **βF** and 407.5 (O_2_) and 377.1 (O_2_) kJ/mol for **αF** while for C sites are 401.4 (C_4_) and 351.3 (C_3_) kJ/mol for **βG**, 398.2 (C_6_) and 368.7 (C_6_) kJ/mol for **αG**, 394.9 (C_6_) and 365.1 (C_6_) kJ/mol for **βF** and 405.3 (C_4_) and 372.2 (C_4_) kJ/mol for **αF**, respectively. The radical structure of **αG** at C_6_ site has been identified before^32^, and this coincides well with the present results that **αGrC**_**6**_ has the lowest Δ*H*_r_ and Δ*G*_r_ values. The comprehensive thermodynamic parameters given in Table 2 can be referenced for subsequent experimental measurements. Solvent effects are further included for the calculations of Δ*H* and Δ*G*, and it shows that these thermodynamic parameters are affected slightly by addition of water solvent (Tables 2 and S6).

### Radical Catalysis

As discussed earlier, for each sugar (**βG**, **αG**, **βF** and **αF**), the most preferential site for radicalization is always at C sites; that is, radicalization is particular by offering a route to activate the C-H bonds in sugars, in contrast to previous attempts focusing on catalysis of O sites. In addition, the Δ*E*_r_ data of C sites in D-glucopyranose and D-fructofuranose conformers are close to each other suggesting a comparable reactivity, and this provides insightful clues to the direct catalytic transformation of D-glucose instead of using D-fructose as the immediate product as widely recommended^2^,^7^,^8^,^9^,^10^,^11^,^12^,^13^,^19^.

**Figure 6:**
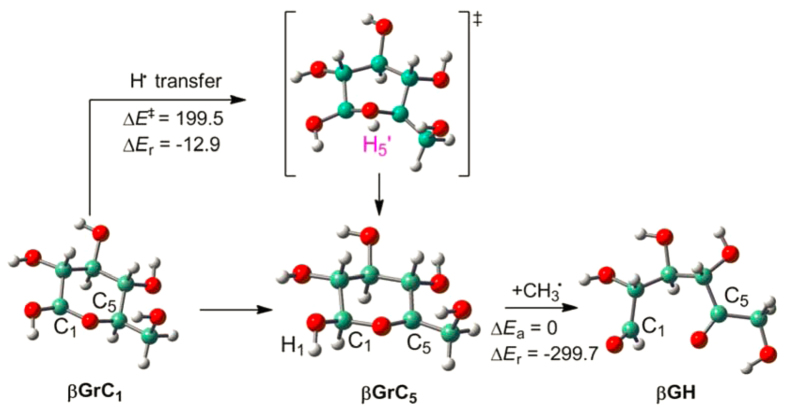
Illustration of catalytic transformation of C-centered sugar radicals. Energy calculations (kJ/mol) are reported at MP2/bs4//B3LYP/bs2 level. Deprivation of H radical (H^•^) is realized with the reaction of **βGrC**_**5**_ + CH_3_^•^ → **βGH **+ CH_4_.

Thermodynamic calculations in Section 3.3 require structural optimizations at MP2/bs4 level that is computationally costly and seems not appropriate for catalytic studies. Here an alternative method is used: two-layer ONIOM(MP2/bs4//M06L/bs3) energies are calculated on basis of B3LYP/bs2 optimized structures. Tables S1, S5 and Figures S3 and S4 show that the alternative obtains comparable Δ*E*_r_ and *RSE* data as the method in Section 2; in addition, this methodology can be safely applied for the accurate energy calculations of other sugar and larger catalytic systems. Figure 6 gives an example for the direct radical transformation of sugars. The conversion from **βGrC**_**1**_ to **βGrC**_**5**_ through H-radical (H^•^) transfer requires an overwhelmingly large activation barrier (Δ*E*_a_ = 199.5 kJ/mol) that is difficult to proceed at ambient conditions; however, the direct radical transformation of **βGrC**_**5**_ is facile, and the H^•^-deprivation reaction (**βGrC**_**5**_ + CH_3_^•^ → **βGH** + CH_4_) is barrierless, which is further driven by the high exothermicity (Δ*E*_r_ = −299.7 kJ/mol). In **βGH**, the pyranyl ring is opened with emergence of two functional groups (−C_1_H = O and −C_5_ = O) that are ready for the future chemical synthesis.

**Figure 7:**
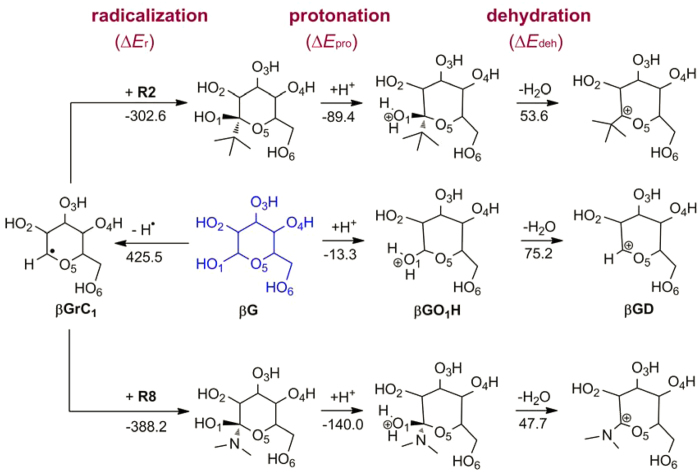
Regulation of catalytic effects of sugars by radical functionalization. Energy calculations (kJ/mol) are reported at ONIOM(MP2/bs4:M06L/bs3)//B3LYP/bs2 level. High-level regions are displayed in ball and stick while low-level regions in stick.

A variety of functional groups can be implanted by radical catalysis^26^,^58^, see Table 3. The reaction energies of **R1**~**R8** with **βGrC**_**1**_ (Δ*E*_r_) are calculated at ONIOM(MP2/bs4//M06L/bs3)//B3LYP/bs2 level and affected pronouncedly by the radical stabilities: Δ*E*_r_ generally has the opposite trend as *RSE* (Table 3). Functionalized sugars are then combined with Brønsted acids to drive cellulosic sugars to downstream products. For the Brønsted-acid catalysis of D-glucopyranose, the initial protonation and dehydration steps are considered to be rate-determining^16^,^59^, and these two steps are depicted in Fig. 7. In line with the previous work^16^, the Brønsted acid is modeled as Zundel complex H_5_O_2_^+^ (an approximation of proton in water), and hence the protonation energy (Δ*E*_pro_) is defined as,


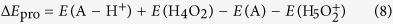


With regard to **βG**, the protonation (Δ*E*_pro_) and dehydration (Δ*E*_deh_) energies are equal to −13.3 and 75.2 kJ/mol, respectively. As indicated in Table 3 and Fig. 7, both of Δ*E*_pro_ and Δ*E*_deh_, especially the former, are affected significantly by functionalization; e.g., the protonation step becomes very favourable for **R8** (H_2_N^•^, Δ*E*_pro_ = −140.0 kJ/mol), while **R7** (Cl^•^) causes this step to be unfavourable (Δ*E*_pro_ = 10.8 kJ/mol). That is, the catalytic effects of cellulosic sugars can be fundamentally regulated by functionalization.

**Figure 8:**
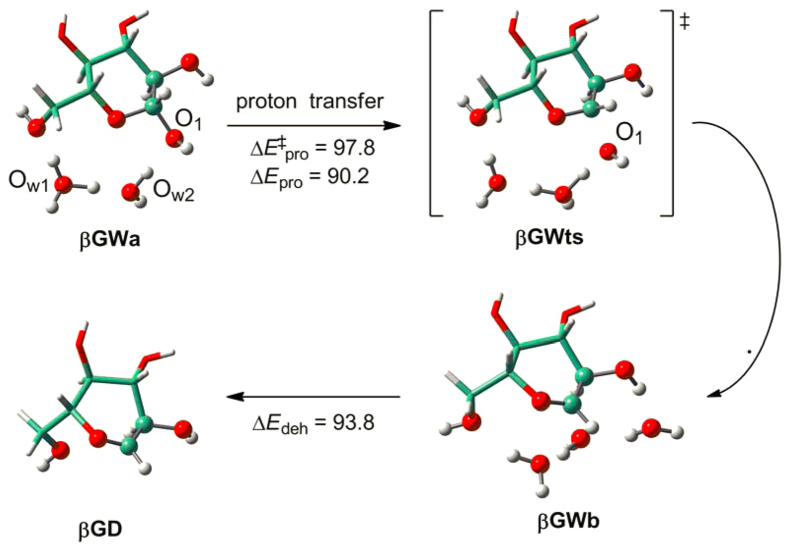
Protonation and dehydration of the O_1_ site in β-D-glucopyranose (βG) in presence of explicit water molecules. Energy calculations (kJ/mol) are reported at ONIOM(MP2/bs4:M06L/bs3)//B3LYP/bs2 level. High-level regions are displayed in ball and stick while low-level regions in stick.

Figure 8 shows the protonation (Δ*E*_pro_) and dehydration (Δ*E*_deh_) processes of **βG** in presence of two explicit water molecules. Protonation of **βG** at O_1_ site causes the formation of one additional water molecule, and this step is thermodynamically unfavorable (Δ*E*_pro_ = 90.2 kJ/mol) with a moderate activation barrier (Δ*E*^‡^_pro_ = 97.8 kJ/mol). The protonation and dehydration processes of **βG** with different functional groups (**R1**~**R8**) are also calculated and the results are given in Table 4 and Figures S5–S12. It indicates that functionalization affects significantly the activation barriers and reaction energies of the protonation steps. For **R5**, O_1_H constructs strong H-bonding with the acetyl O atom (1.772 Å, see Figure S9), and this adds the difficulty of protonation resulting in a considerably large activation barrier (Δ*E*^‡^_pro_ = 131.3 kJ/mol). The protonation thermodynamics can be significantly driven by the functional groups with more steric hindrances (e.g., **R2**, **R4 **> **R1**, **R3 **> H^•^ in **βG**) or by the high stabilities of protonation products (**R6** and **R8**). In the case of **R6**, the protonation product is stabilized pronouncedly by two strong H-bonds (O_2_H_2_…O_1_: 2.062 Å and O_7_H_7_…O_1_: 1.469 Å, see Figure S10). In contrast, the reaction energies of dehydration seem not to be much affected by different functional groups, which are generally around 90.0 kJ/mol except **R6** corresponding to the particularly stable protonation product (Δ*E*_deh_ = 117.9 kJ/mol).

## Additional Information

**How to cite this article**: Yang, G. *et al*. Radicalization and Radical Catalysis of Biomass Sugars: Insights from First-principles Studies. *Sci. Rep.* **6**, 29711; doi: 10.1038/srep29711 (2016).

## Supplementary Material

Supplementary Information

## Figures and Tables

**Table 1:** MP2/bs4 calculated radical generation energies (Δ*E*_r_) for D-glucopyranose and D-fructofuranose conformers at the various O/C sites^a^^,^^b^.

	βG		αG		βF		αF	
	O	C	O	C	O	C	O	C
C_1_/O_1_	**495.5**	**427.3**	**500.5**	441.1	496.7	429.2	494.3	440.1
C_2_/O_2_	505.9	438.0	503.8	443.9	516.6		**436.4**	
C_3_/O_3_	506.0	429.2	506.6	431.5	505.7	430.0	500.4	440.7
C_4_/O_4_	506.5	427.7	510.7	431.1	497.4	428.0	504.6	**432.4**
C_5_/O_5_		439.8		443.1		435.1		434.1
C_6_/O_6_	495.8	428.6	503.1	**425.3**	502.4	**422.4**	494.5	434.7
Average	501.9	431.8	504.9	436.0	503.8	428.9	486.0	436.4

^a^Energy units in kJ/mol.

^b^In each case, the lowest radical generation energy has been highlighted in bold.

**Table 2:** Enthalpy changes (Δ*H*_r_) and Gibbs free energies (Δ*G*_r_) for the radicalization of D-glucopyranose and D-fructofuranose conformers^a^^,^^b^.

	βG		αG		βF		αF	
	Δ*H*_r_	Δ*G*_r_	Δ*H*_r_	Δ*G*_r_	Δ*H*_r_	Δ*G*_r_	Δ*H*_r_	Δ*G*_r_
O_1_	466.1	**381.2**	470.0	**440.8**	466.4	**437.2**	464.0	435.1
O_2_	477.5	399.3	473.5	443.8	486.7	455.7	407.5	**377.1**
O_3_	476.3	399.8	476.1	446.9	477.4	449.4	471.5	442.2
O_4_	476.8	401.7	482.2	453.7	469.7	439.1	475.6	442.9
O_6_	465.5	392.7	472.4	441.4	471.0	440.0	463.5	434.9
C_1_	401.8	353.0	413.8	382.5	401.7	370.4	412.1	380.1
C_2_	411.6	360.3	416.2	381.8				
C_3_	403.1	**351.3**	405.1	374.1	403.6	373.4	413.6	378.2
C_4_	401.4	355.7	404.8	373.9	401.3	371.7	405.3	**372.2**
C_5_	413.9	358.7	415.6	380.6	408.1	373.7	407.4	374.6
C_6_	401.9	359.0	398.2	**368.7**	394.9	**365.1**	407.0	377.0

^a^Energy units in kJ/mol.

^b^In each case, the lowest Δ*G*_r_ value has been highlighted in bold.

**Table 3:** ONIOM(MP2/bs4//M06L/bs3)//B3LYP/bs2 calculated energies for the radical catalysis of βGrC_1_^a^^,^^b^.

No.	Chemical formula	*RSE*	Δ*E*_r_	Δ*E*_pro_	Δ*E*_deh_
				−13.3	75.2
**R1**	H_3_C^•^	0	−381.0	−52.9	51.7
**R2**	(CH_3_)_3_C^•^	−55.4	−302.6	−89.4	53.6
**R3**	CH_2_ = CHCH_2_^•^	−84.2	−295.7	−53.5	47.0
**R4**	C_6_H_5_^•^ (phenyl)	10.9	−399.5	−85.8	46.7
**R5**	CH_3_C^•^ = O (acetyl)	−77.8	−325.9	−44.6	91.8
**R6**	OH^•^	35.3	−439.2	−46.4	31.2
**R7**	Cl^•^	−24.0	−364.2	10.8	52.4
**R8**	H_2_N^•^	−1.7	−388.2	−140.0	47.7

^a^Energy units in kJ/mol.

^b^Data of the first line are for βG.

**Table 4:** ONIOM(MP2/bs4//M06L/bs3)//B3LYP/bs2 calculated energies for the radical catalysis of βGrC_1_ in presence of two water molecules^a^^,^^b^.

No.	chemical formula	Δ*E*^‡^_pro_	Δ*E*_pro_	Δ*E*_deh_
		97.8	90.2	93.8
**R1**	H_3_C^•^	80.0	36.2	92.0
**R2**	(CH_3_)_3_C^•^	35.5	15.5	84.7
**R3**	CH_2_ = CHCH_2_^•^	84.4	46.5	85.3
**R4**	C_6_H_5_^•^ (phenyl)	88.8	13.2	81.9
**R5**	CH_3_C^•^ = O (acetyl)	131.3	90.3	83.9
**R6**	OH^•^	74.1	0.9	117.9
**R7**	Cl^•^	111.9	84.6	93.0
**R8**	H_2_N^•^	30.9	−55.7	97.6

^a^Energy units in kJ/mol.

^b^Data of the first line are for βG.
