# Supplementary material for: Radicalization and Radical Catalysis of Biomass Sugars: Insights from First-principles Studies
Source: Sci Rep. 2016 Jul 13;6:29711. doi: 10.1038/srep29711 (PMC4942814; doi:10.1038/srep29711)
Supplement: Supplementary Information [file srep29711-s1.pdf]

Supporting Information for:

Radicalization and Radical Catalysis of Biomass Sugars: Insights from First-principles Studies

Gang Yang*, Chang Zhu, Xianli Zou, Lijun Zhou

College of Resources and Environments & Chongqing Key Laboratory of Soil Multi-scale Interfacial Process, Southwest University, Chongqing 400715, China

Table S1. Averages and standard deviations (*S.D.*) of radical generation energy deviations (<δΔ*E*>) for β-D-glucopyranose (**βG**) calculated by other methodologies indicated below vs. MP2/bs4 level[a]

	**O**		**C**	
	<δΔ*E*>	*S.D.*	<δΔ*E*>	*S.D.*
HF/bs4	161.7	2.3	100.2	3.2
PBE1PBE/bs4	47.6	4.4	24.5	3.0
BP86/bs4	-18.1	6.2	-39.7	3.2
B3LYP/bs4	45.1	5.5	18.9	3.7
M06L/bs4	59.9	9.5	21.6	3.4
B3LYP/bs1	64.7	6.4	14.9	3.1
B3LYP/bs2	43.4	5.0	13.4	3.5
B3LYP/bs3	40.3	5.2	12.5	3.5
MP2/bs4//B3LYP/bs1	-2.0	2.1	-1.1	2.4
MP2/bs4//B3LYP/bs2	-1.9	2.0	-1.1	2.3
MP2/bs4//B3LYP/bs3	-2.2	2.3	-1.1	2.3
MP2/bs4//B3LYP/bs4	-2.4	2.4	-1.2	2.2
ONIOM(MP2/bs4//M06L /bs3)//B3LYP/bs2	-0.1	1.8	0.2	2.0

[a] Energy units in kJ/mol.

Table S2. Enthalpy changes (ΔH_r) and Gibbs free energies (ΔG_r) for the radicalization of various molecules[a]

	B3LYP/bs2		MP2/bs4//B3LYP/bs2[b]		MP2/bs4[b,c]		MP2/bs4	
	ΔH_r	ΔG_r	ΔH_r	ΔG_r	ΔH_r	ΔG_r	ΔH_r	ΔG_r
H_2O	484.6	453.6	505.7	474.7	505.8	474.7	506.0	475.0
CH_4	438.2	403.2	432.4	397.5	432.4	397.4	431.9	396.9
$CH_3O\underline{\mathbf{H}}$[d]	418.2	384.4	455.0	421.1	454.7	420.9	456.1	422.6
$C\underline{\mathbf{H}}_3OH$[d]	398.6	363.8	399.3	364.5	399.3	364.5	398.8	364.4
$CH_3CH_2O\underline{\mathbf{H}}$[d]	416.9	380.8	460.0	421.1	456.6	420.5	458.2	423.9
$CH_3C\underline{\mathbf{H}}_2OH$[d]	389.6	353.8	395.3	359.5	395.3	359.4	395.2	359.6
$C\underline{\mathbf{H}}_3CH_2OH$[d]	425.0	386.9	426.5	388.4	426.4	388.4	426.5	388.6
$CH_2OHCH_2O\underline{\mathbf{H}}$[d]	420.9	386.9	460.6	426.7	460.2	426.3	461.7	427.7
$CH_2OHC\underline{\mathbf{H}}_2OH$[d]	393.5	356.9	401.4	364.8	401.2	364.6	401.3	364.9
$C\underline{\mathbf{H}}_2OHCH_2OH$[d]	391.3	356.2	398.4	363.3	398.3	363.3	398.4	363.3

[a] Energy units in kJ/mol;

[b] Frequency calculations at B3LYP/bs2 level of theory;

[c] Default methodology for thermodynamic calculations;

[d] H atom of being radicalized is underlined and in bold.

Table S3. Deviations of enthalpy changes (ΔH_r) and Gibbs free energies (ΔG_r) of other methodologies vs. MP2/bs4 for the radicalization of various molecules[a]

	B3LYP/bs2		MP2/bs4//B3LYP/bs2[b]		MP2/bs4[b,c]	
	$\delta\Delta H_r$	$\delta\Delta G_r$	$\delta\Delta H_r$	$\delta\Delta G_r$	$\delta\Delta H_r$	$\delta\Delta G_r$
H_2O	-21.4	-21.4	-0.3	-0.3	-0.2	-0.3
CH_4	6.3	6.3	0.5	0.6	0.5	0.5
CH_3O**H**[d]	-37.9	-38.2	-1.1	-1.5	-1.4	-1.7
C**H**$_3$OH[d]	-0.2	-0.6	0.5	0.1	0.5	0.1
CH_3CH_2O**H**[d]	-41.3	-43.1	1.8	-2.8	-1.6	-3.4
CH_3C**H**$_2$OH[d]	-5.6	-5.8	0.1	-0.1	0.1	-0.2
C**H**$_3CH_2OH$[d]	-1.5	-1.7	0	-0.2	-0.1	-0.2
CH_2OHCH_2O**H**[d]	-40.8	-40.8	-1.1	-1.0	-1.5	-1.4
CH_2OHC**H**$_2$OH[d]	-7.8	-8.0	0.1	-0.1	-0.1	-0.3
C**H**$_2OHCH_2OH$[d]	-7.1	-7.1	0	0	-0.1	0

[a] Energy units in kJ/mol;

[b] Frequency calculations at B3LYP/bs2 level of theory;

[c] Default methodology for thermodynamic calculations;

[d] H atom of being radicalized is underlined and in bold.

Table S4. MP2/bs4 calculated radical stabilization energies (*RSE*) for D-glucopyranose and D-fructofuranose conformers at the various O/C sites[a,b]

	βG		αG		βF		αF	
	O	C	O	C	O	C	O	C
C_1/O_1	**33.0**	**-35.2**	**38.0**	-21.4	31.9	-22.4	**34.2**	-33.3
C_2/O_2	43.5	-24.4	41.3	-18.6				
C_3/O_3	43.5	-33.2	44.1	-31.0	**-26.1**	-21.8	54.1	-32.5
C_4/O_4	44.0	-34.8	48.2	-31.4	37.9	**-30.1**	43.2	-34.5
C_5/O_5		-22.7		-19.4	42.2	-28.4	34.9	-27.4
C_6/O_6	33.3	-33.8	40.6	**-37.2**	32.0	-27.8	39.9	**-40.1**

[a] Energy units in kJ/mol;

[b] In each case, the lowest radical stabilization energy has been highlighted in bold.

Table S5. Radical generation energies (ΔE_r) and stabilization energies (*RSE*) for β-D-glucopyranose (**βG**) at two theoretical levels as well as their deviations ($\delta\Delta E_r$ and δRSE) from the data of the default methodology[a]

	B3LYP/bs2				ONIOM(MP2/bs4//M06L/bs3)//B3LYP/bs2			
	ΔE_r	$\delta\Delta E_r$	*RSE*	δRSE	ΔE_r	$\delta\Delta E_r$	*RSE*	δRSE
O_1	443.8	-51.7	-24.5	-57.5	498.3	2.8	35.8	2.8
O_2	461.8	-44.1	-6.4	-49.9	503.3	-2.6	40.8	-2.6
O_3	464.7	-41.3	-3.6	-47.1	506.4	0.5	43.9	0.5
O_4	465.4	-41.1	-2.9	-46.9	506.2	-0.3	43.7	-0.3
O_6	457.1	-38.7	-11.2	-44.5	496.4	0.6	33.9	0.6
C_1	414.1	-13.2	-54.2	-19.0	425.7	-1.6	-36.8	-1.6
C_2	422.9	-15.1	-45.4	-20.9	437.8	-0.2	-24.7	-0.2
C_3	413.4	-15.8	-54.8	-21.6	429.0	-0.2	-33.5	-0.2
C_4	417.5	-10.2	-50.8	-16.0	431.1	3.5	-31.4	3.4
C_5	422.0	-17.7	-46.2	-23.5	438.8	-1.0	-23.7	-1.0
C_6	422.1	-8.5	-48.1	-14.3	427.8	-0.8	-34.6762	-0.8

[a] Energy units in kJ/mol.

Table S6. Enthalpy changes (ΔH_r) and Gibbs free energies (ΔG_r) for the radicalization of solvated D-glucopyranose and D-fructofuranose conformers, where solvent effects are accounted for by PCM solvation model[a,b]

	**βG**		**αG**		**βF**		**αF**	
	H_r	G_r	H_r	G_r	H_r	G_r	H_r	G_r
O_1	465.6	**380.8**	470.6	441.4	468.0	438.8	467.0	438.1
O_2	475.3	397.2	471.8	442.1	485.3	454.2	402.7	**372.3**
O_3	472.8	396.3	471.4	442.2	471.5	443.6	475.8	446.5
O_4	471.7	396.7	476.5	447.9	467.0	**436.4**	469.0	436.3
O_6	466.0	393.3	467.9	**438.0**	469.6	438.6	466.6	438.0
C_1	404.5	355.7	415.5	384.2	401.5	370.2	409.6	377.6
C_2	409.8	358.6	415.5	381.1				
C_3	403.6	**351.9**	404.9	373.9	403.7	373.5	408.3	372.9
C_4	399.3	353.7	406.1	375.2	400.2	370.6	403.3	**370.2**
C_5	411.9	356.7	412.8	377.8	403.8	369.4	406.5	373.7
C_6	403.9	361.0	400.2	**370.7**	395.6	**365.9**	410.8	380.7

[a] Energy units in kJ/mol;

[b] In each case, the lowest ΔG_r value has been highlighted in bold.

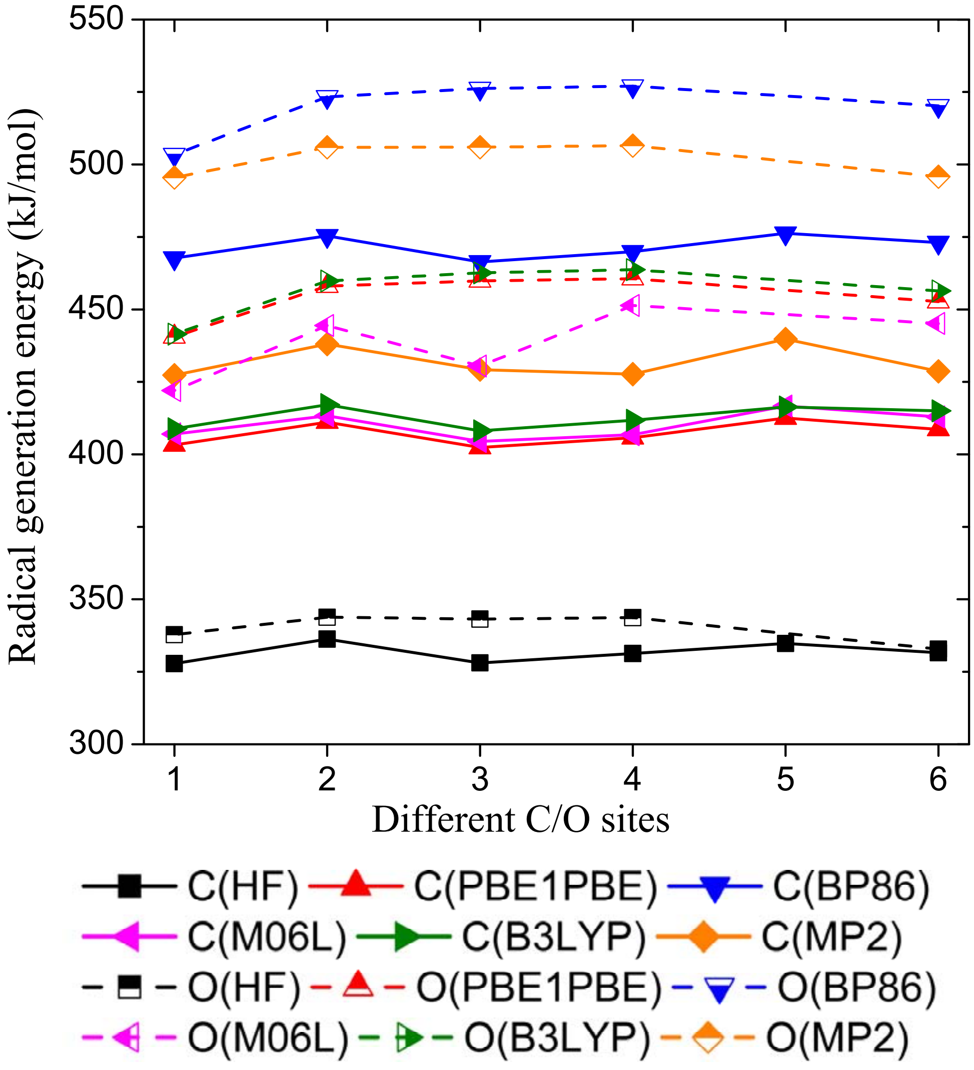

Figure S1. Radical generation energies of β-D-glucopyranose (**βG**) obtained by different theoretical methods (basis set: bs4).

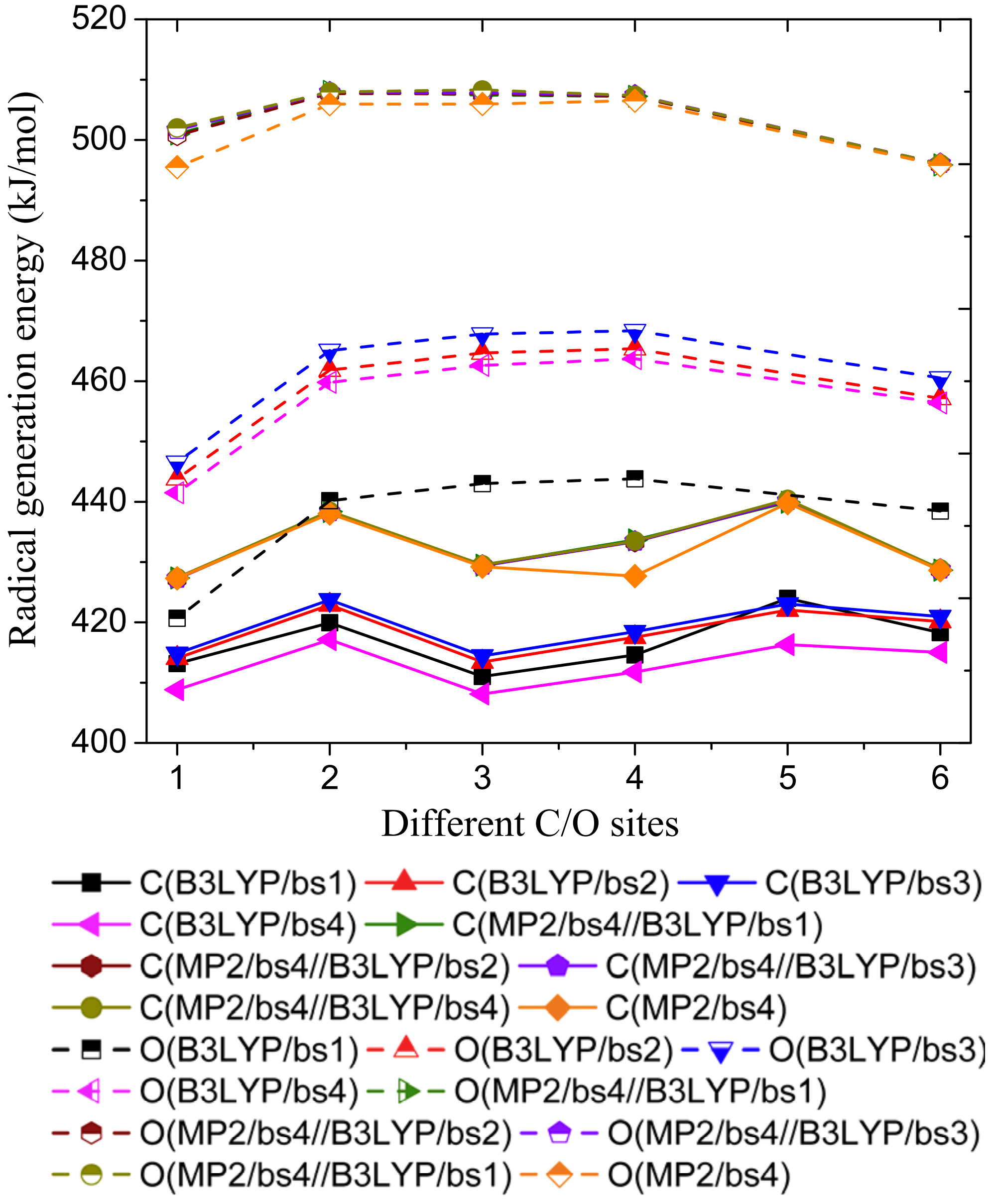

Figure S2. Radical generation energies of β-D-glucopyranose (**βG**) obtained at the various levels of theory.

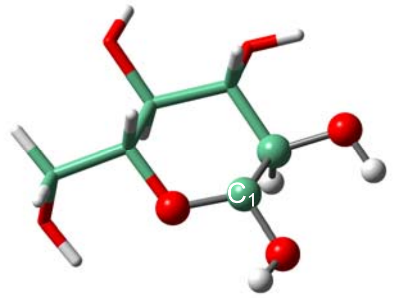

βGrC$_1$

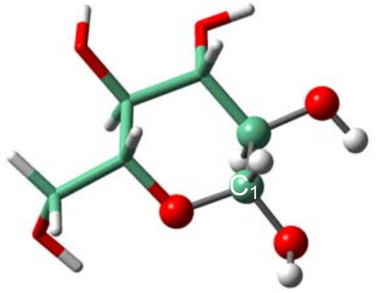

βG (C$_1$)

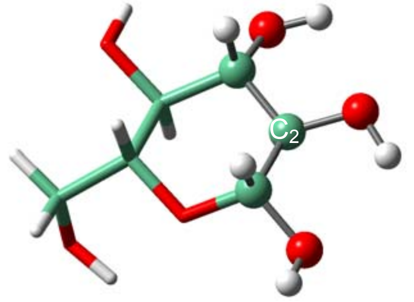

βGrC$_2$

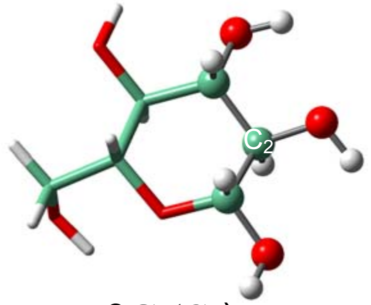

βG (C$_2$)

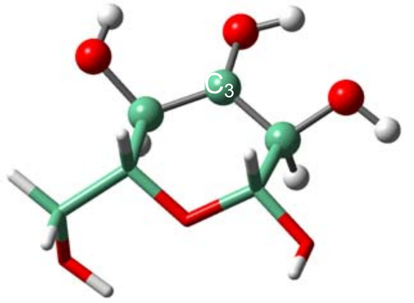

βGrC$_3$

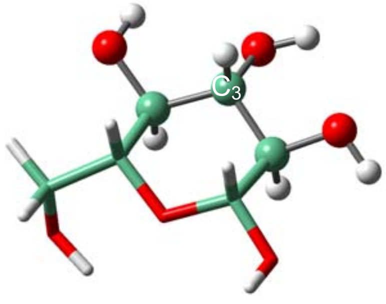

βG (C$_3$)

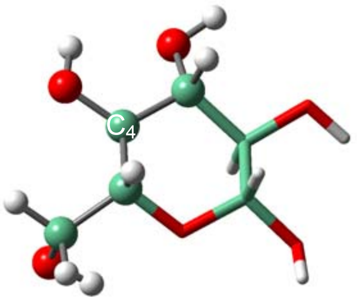

βGrC$_4$

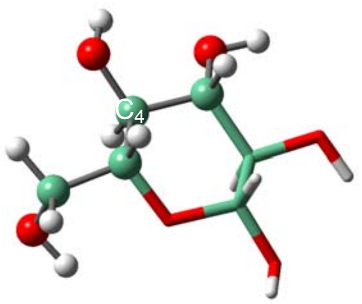

βG (C$_4$)

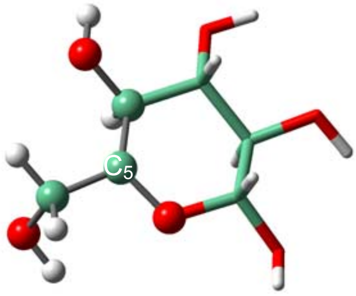

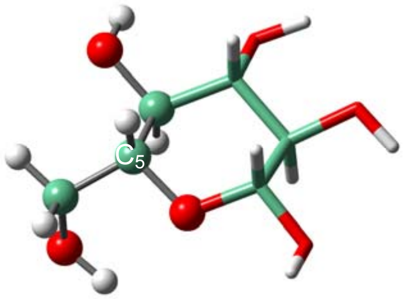

βGrC$_5$ **βG** (C_5)

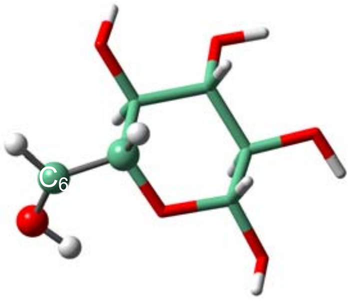

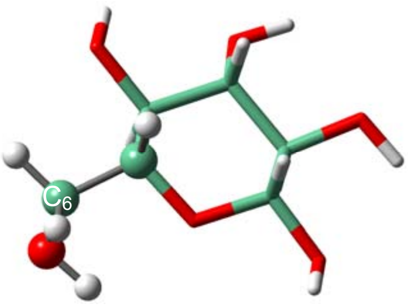

βGrC$_6$ **βG** (C_6)

Figure S3. Two-layer ONIOM methodology to calculate the radical generation energies (ΔE_r) and stabilization energies (*RSE*) corresponding to the various C sites. High-level regions are displayed in ball and stick while low-level regions in stick.

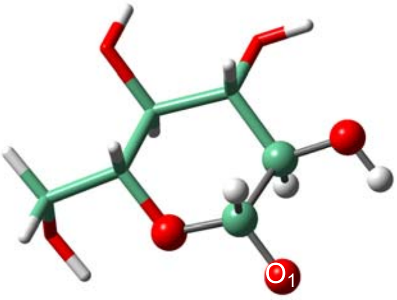

βGrO$_1$

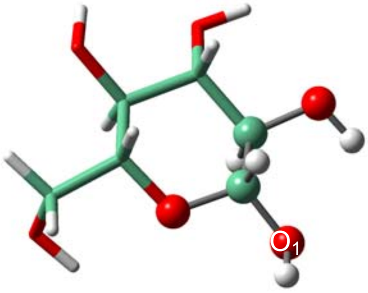

βG (O$_1$)

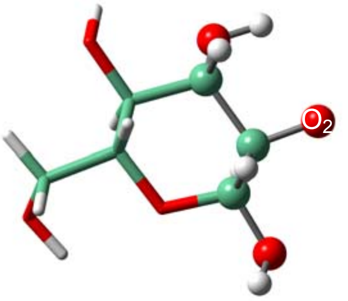

βGrO$_2$

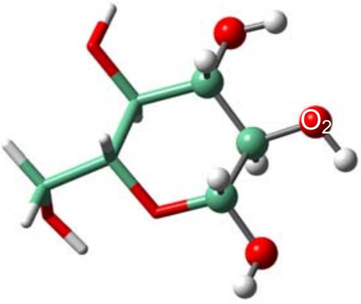

βG (O$_2$)

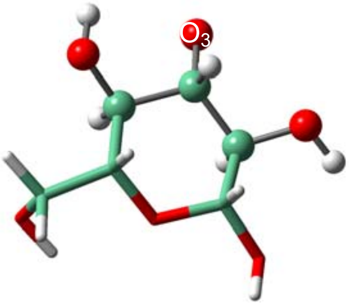

βGrO$_3$

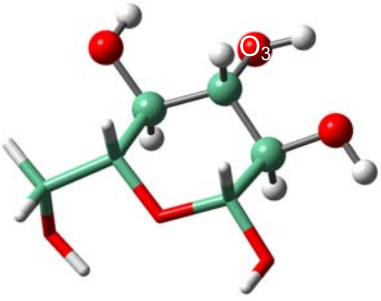

βG (O$_3$)

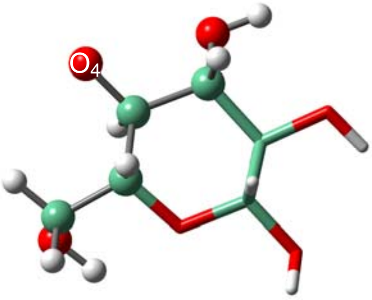

βGrO$_4$

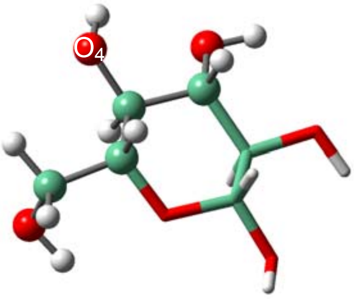

βG (O$_4$)

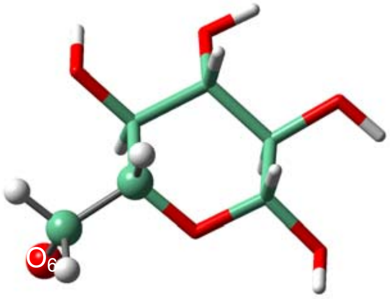

βGrO$_6$

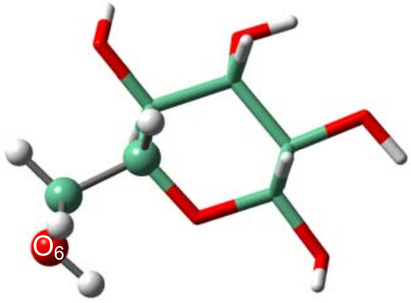

βG (O$_6$)

Figure S4. Two-layer ONIOM methodology to calculate the radical generation energies (ΔE_r) and stabilization energies (*RSE*) corresponding to the various O sites. High-level regions are displayed in ball and stick while low-level regions in stick.

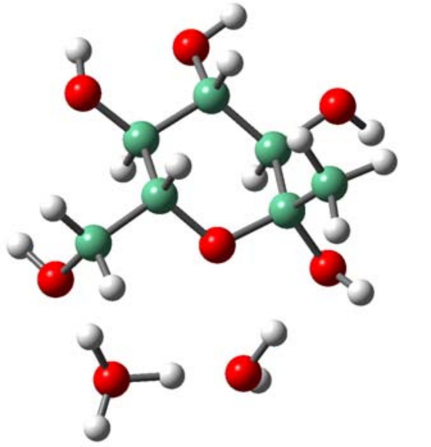

βGR1Wa

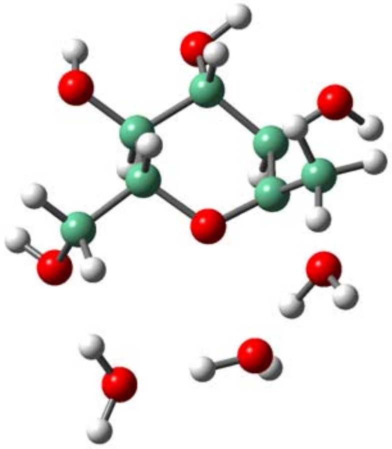

βGR1Wts

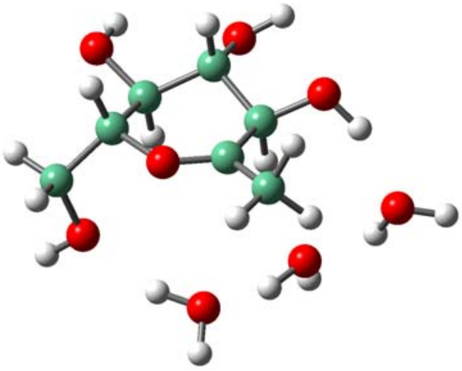

βGR1Wb

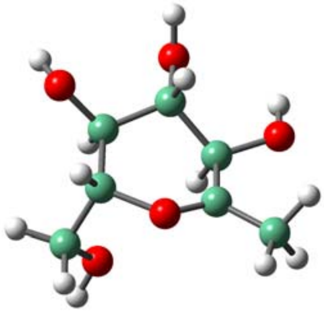

βGR1D

Figure S5. Structures of protonation and dehydration processes for the O_1 site of functionalized β-D-glucopyranose (**βG**, **R1** = $H_3C^{\bullet}$) in presence of explicit water molecules.

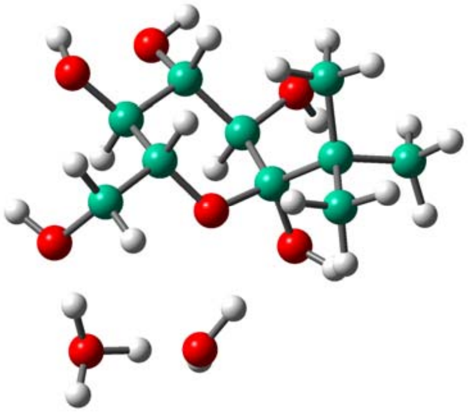

βGR2Wa

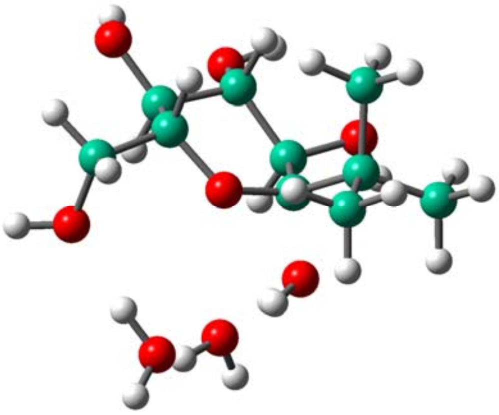

βGR2Wts

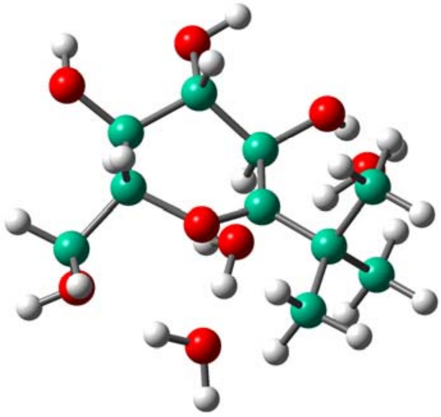

βGR2Wb

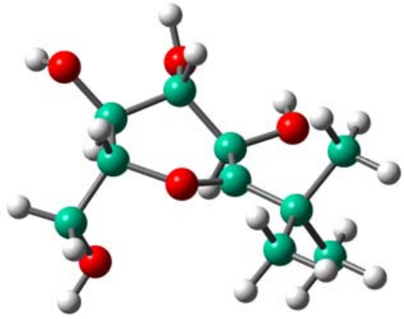

βGR2D

Figure S6. Structures of protonation and dehydration processes for the O_1 site of functionalized β-D-glucopyranose (**βG**, **R2** = $(CH_3)_3C^{\bullet}$) in presence of explicit water molecules.

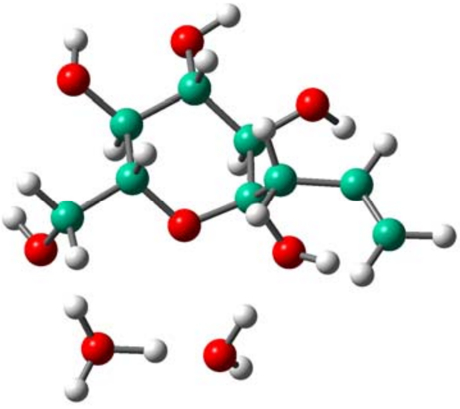

βGR3Wa

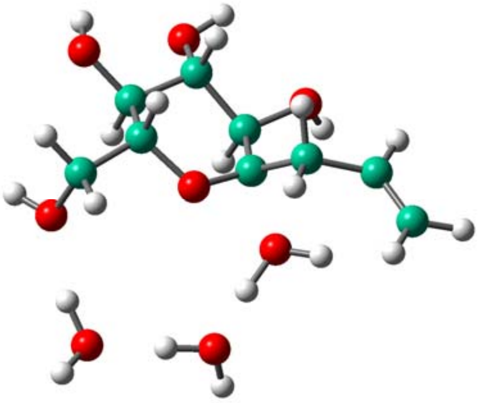

βGR3Wts

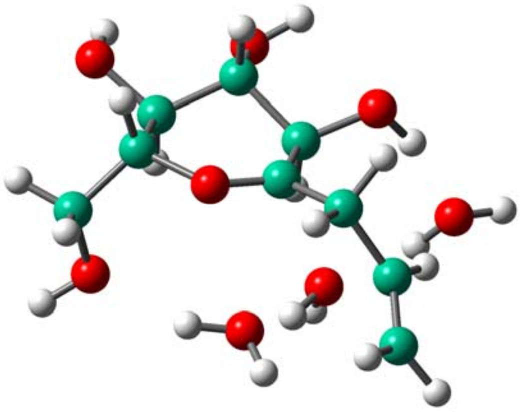

βGR3Wb

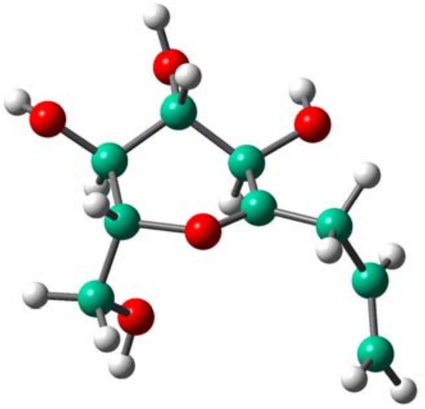

βGR3D

Figure S7. Structures of protonation and dehydration processes for the O_1 site of functionalized β-D-glucopyranose (**βG**, **R3** = $CH_2=CHCH_2^{\bullet}$) in presence of explicit water molecules.

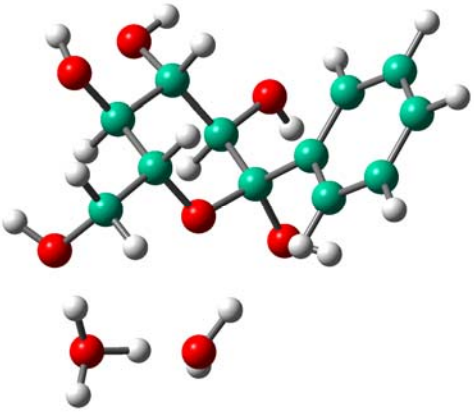

βGR4Wa

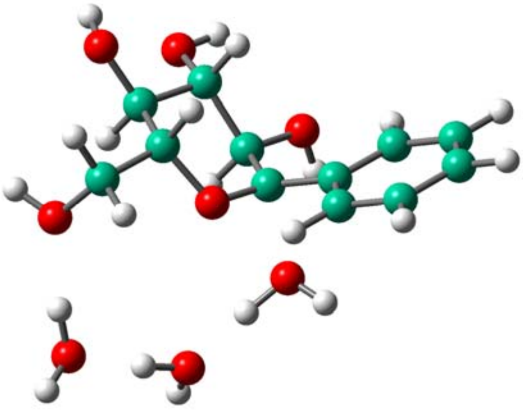

βGR4Wts

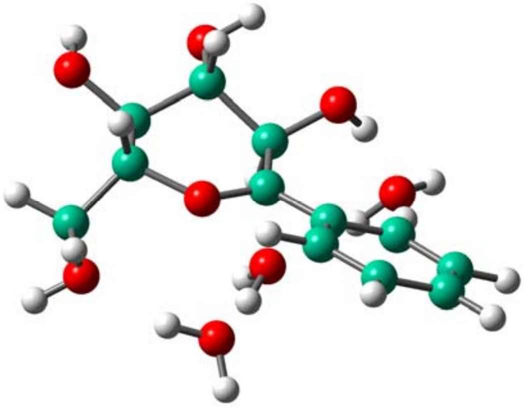

βGR4Wb

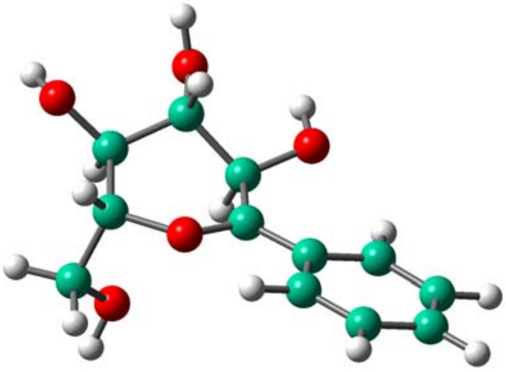

βGR4D

Figure S8. Structures of protonation and dehydration processes for the O_1 site of functionalized β-D-glucopyranose (**βG**, **R4** = $C_6H_5^{\bullet}$) in presence of explicit water molecules.

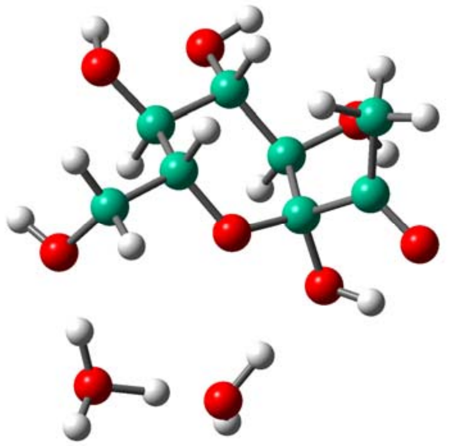

βGR5Wa

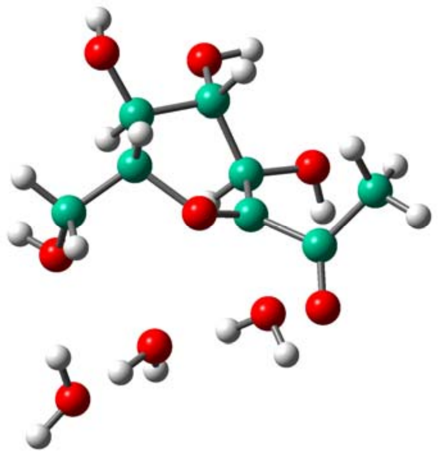

βGR5Wts

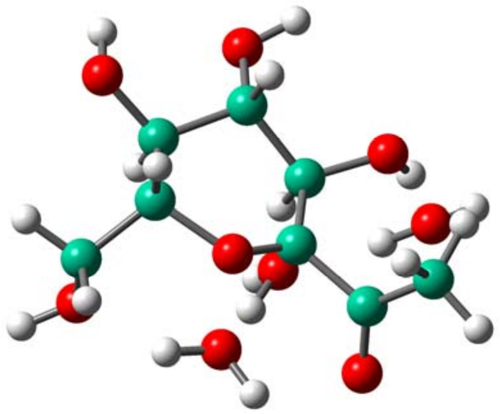

βGR5Wb

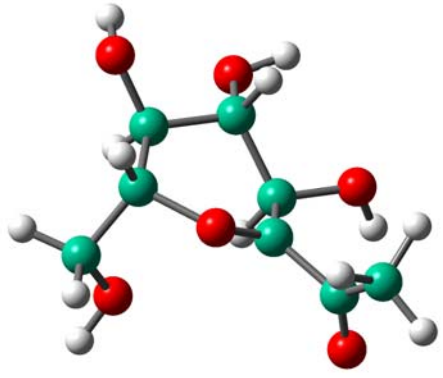

βGR5D

Figure S9. Structures of protonation and dehydration processes for the O_1 site of functionalized β-D-glucopyranose (**βG**, **R5** = $CH_3C^{\bullet}{=}O$) in presence of explicit water molecules.

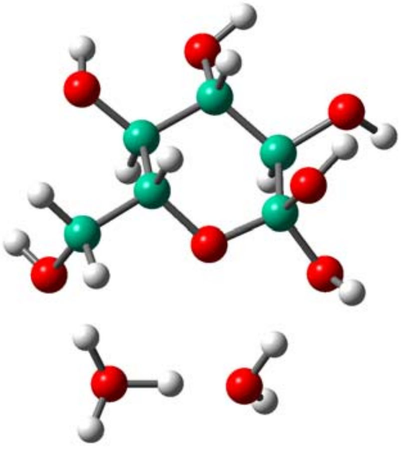

βGR6Wa

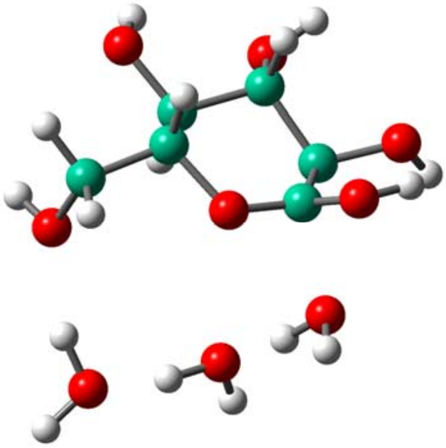

βGR6Wts

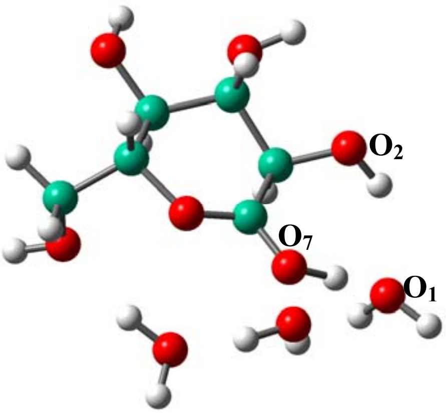

βGR6Wb

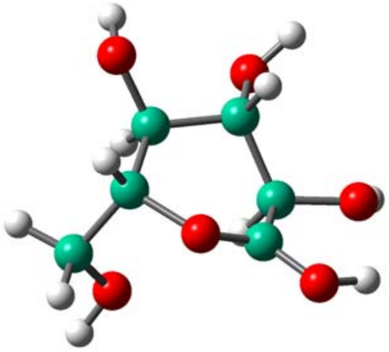

βGR6D

Figure S10. Structures of protonation and dehydration processes for the O_1 site of functionalized β-D-glucopyranose (**βG**, **R6** = OH$^{\bullet}$) in presence of explicit water molecules.

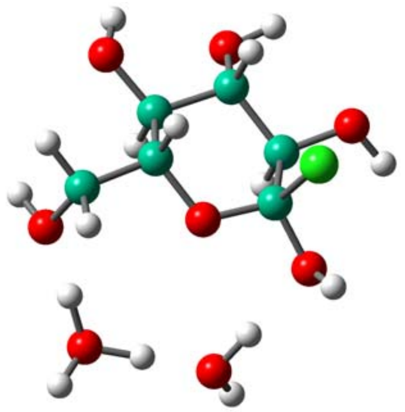

βGR7Wa

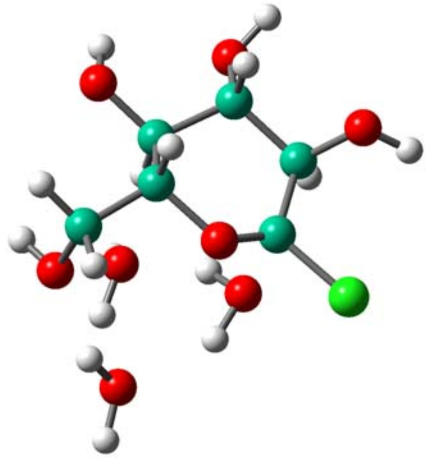

βGR7Wts

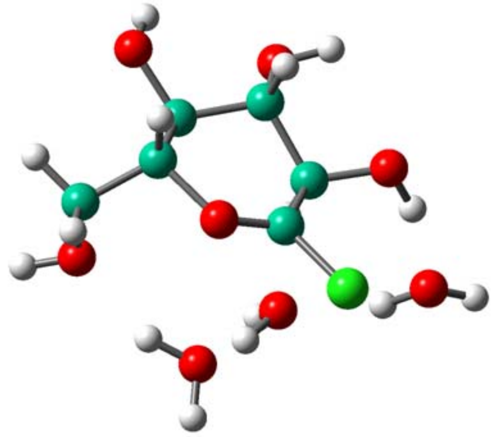

βGR7Wb

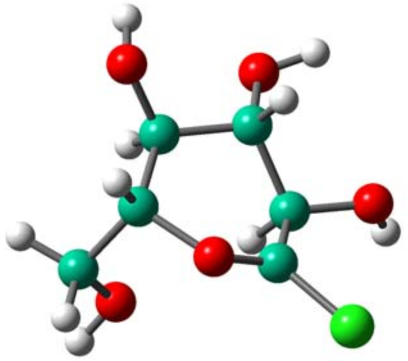

βGR7D

Figure S11. Structures of protonation and dehydration processes for the O_1 site of functionalized β-D-glucopyranose (**βG**, **R7** = $Cl^{\bullet}$) in presence of explicit water molecules.

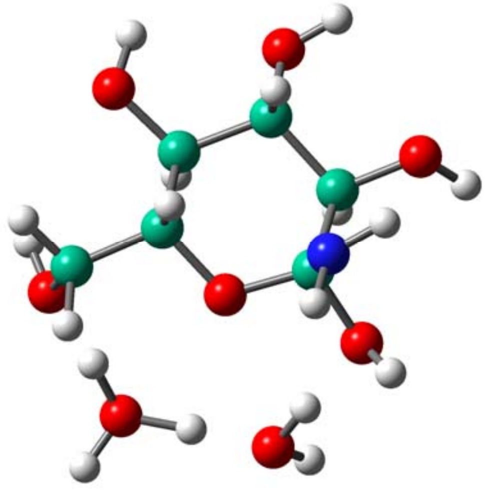

βGR8Wa

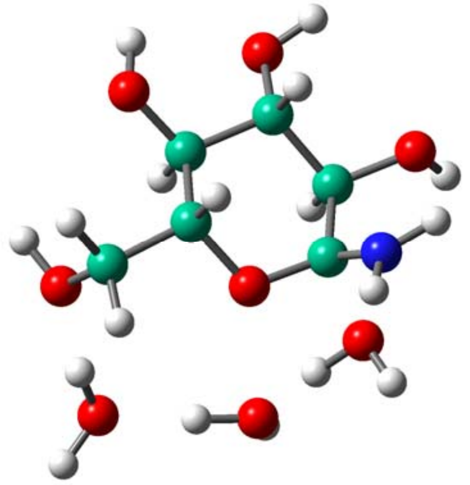

βGR8Wts

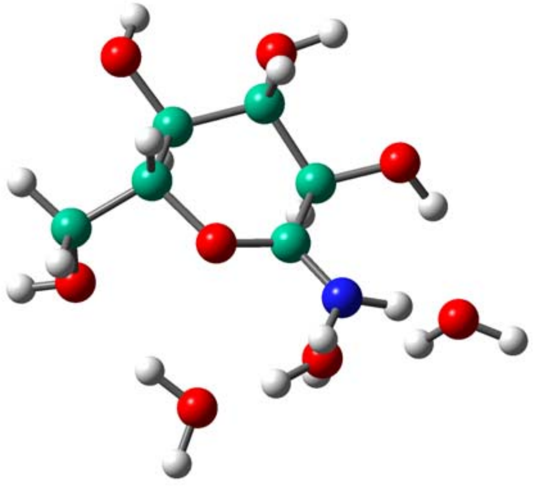

βGR8Wb

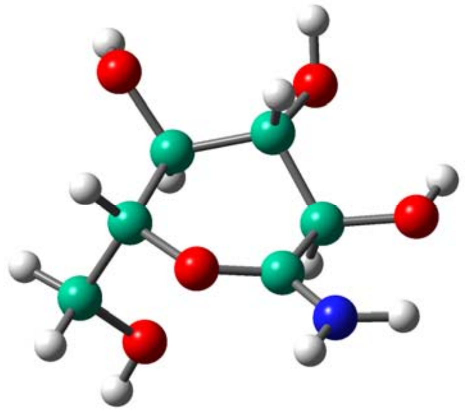

βGR8D

Figure S12. Structures of protonation and dehydration processes for the O_1 site of functionalized β-D-glucopyranose (**βG**, **R8** = $H_2N^{\bullet}$) in presence of explicit water molecules.
